# Down-Regulated CLDN10 Predicts Favorable Prognosis and Correlates With Immune Infiltration in Gastric Cancer

**DOI:** 10.3389/fgene.2021.747581

**Published:** 2021-10-13

**Authors:** XiongHui Rao, JianLong Jiang, ZhiHao Liang, JianBao Zhang, ZheHong Zhuang, HuaiYu Qiu, Huixing Luo, Nuoqing Weng, Xiaobin Wu

**Affiliations:** ^1^ Department of Gastrointestinal Surgery, The Eighth Affiliated Hospital of Sun Yat-Sen University, Shenzhen, China; ^2^ Department of Gastrointestinal Surgery, The Seventh Affiliated Hospital of Sun Yat-Sen University, Shenzhen, China

**Keywords:** CLDN10, gastric cancer, prognosis, immune-infiltration, biomarker

## Abstract

**Background:** CLDN10, an important component of the tight junctions of epithelial cells, plays a crucial role in a variety of tumors. The effect of CLDN10 expression in gastric cancer, however, has yet to be elucidated.

**Methods:** Differential expression of CLDN10 at the mRNA and protein levels was evaluated using Oncomine, ULCAN, HPA and TIMER2.0 databases. Real-time polymerase chain reaction (RT-PCR) was utilized to further verify the expression of CLDN10 *in vitro*. Correlations between CLDN10 expression and clinical outcomes of gastric cancer were explored by Kaplan-Meier Plotter. Gene set enrichment analysis (GSEA) and protein-protein interaction (PPI) were performed *via* LinkedOmics and GeneMANIA. The correlations between CLDN10 expression and immune cell infiltration and somatic copy number alternations (SCNA) in gastric cancer were explored by TIMER2.0 and GEPIA2.0.

**Results:** CLDN10 expression was lower in gastric cancer compared to adjacent normal tissues, and associated with better prognosis. CLDN10 also showed significant differences at different T stages, Lauren classification, treatments and HER2 status. PPI and GSEA analysis showed that CLDN10 might be involved in signal transmission, transmembrane transport and metabolism. In some major immune cells, low expression of CLDN10 was associated with increased levels of immune cell infiltration. In addition, it was found that different SCNA status in CLDN10 might affect the level of immune cell infiltration. Furthermore, the expression of CLDN10 was significantly associated with the expression of several immune cell markers, especially B cell markers, follicular helper T cell (Tfh) markers and T cell exhaustion markers.

**Conclusion:** Down-regulated CLDN10 was associated with better overall survival (OS) in gastric cancer. And CLDN10 may serve as a potential prognostic biomarker and correlate to immune infiltration levels in gastric cancer.

## Introduction

Gastric cancer is the fifth most common malignant tumor and the third leading cause of cancer-related death in humans, with 1 million new cases diagnosed each year (Bray et al., 2018). Due to the lack of reliable biomarkers and early screening methods, gastric cancer is usually first diagnosed at an advanced stage. As a resultpatients remain at a high risk for metastasis and death despite the continuous advancements that have been achieved in surgery, perioperative chemotherapy, and targeted therapy (Mereiter et al., 2016; Rawla and Barsouk 2019). In recent years, immunotherapy has become a hot topic for tumor treatment. Immune checkpoint inhibitors targeting PD1, CTLA4 and other targets have shown remarkable achievements in combatting malignant melanoma, non-small cell lung cancer, renal cell carcinoma, gastrointestinal tumors and other tumors (Larkin et al., 2015; Kang et al., 2017; Janjigian et al., 2018; Motzer et al., 2018; Overman et al., 2018; Hellmann et al., 2019). Thus, the identification of new biomarkers and therapeutic targets is crucially important for the future treatment of gastric cancer.

Tight junctions, also known as occluding junctions, are one of the important connection forms of cell adhesion structures (Markov 2013). Tight junctions have two main functions (i) they act as a barrier and regulate molecular transport, and (ii) they play a role in maintaining cell polarity. Such a loss of cell polarity is a biological characteristic of cancer cells. When the tight junction function is impaired, its barrier function decreases, leading to increased tissue permeability causing the polarity of endothelial cells to disappear, eventually leading to genetic diseases, immune diseases and even tumors(Sawada et al., 2003; Krause et al., 2008). Claudins are key components of tight junctions, and abnormal expression could promote or inhibit the occurrence and development of tumors, and play an important role in the process of tumor proliferation, invasion, migration and metastasis. Accumulated evidence has proven that claudins could be used as prognostic biomarkers or to screen potential populations for targeted therapy (Singh, Toom, and Huang 2017; Tabariès and Siegel 2017; Sahin et al., 2021). CLDN10, a member of the claudin family, maintains cell-cell adhesion, the loss of which has been considered to be the initial stage of tumor cell migration(Hirohashi and Kanai 2003). Abnormal expression of CLDN10 may cause dysfunction of tight junctions, thereby affecting tumor progression, although this effect is complex. Previous reports have shown that CLDN10 is highly expressed in hepatocellular carcinoma, papillary thyroid carcinoma and lung adenocarcinoma. Its expression is associated with poor prognosis of hepatocellular carcinoma, but with better survival of thyroid papillary carcinoma and lung adenocarcinoma(Huang et al., 2011; Zhang et al., 2013; Xiang et al., 2020). In addition, down-regulated CLDN10 is correlated with poor prognosis of ovarian cancer and clear cell renal cell carcinoma(Li, Xuan, et al., 2020; Yang et al., 2020). Currently, there is a lack of systematic studies to identify the underlying value of CLDN10 in the prognosis and clinicopathological characteristics of gastric cancer.

Here Oncomine, ULCAN, Human Protein Atlas (HPA), Kaplan–Meier plotter and GEPIA 2.0 were used to analyze CLDN10 expression and its correlation with the prognosis and clinicopathological features of gastric cancer. Subsequently the association between CLDN10 and the level of immune cell infiltration in the tumor microenvironment was investigated using TIMER 2.0. Our data revealed an important role of CLDN10 in gastric cancer, and to some extent explains the potential relationship and mechanism of the interaction between CLDN10 and the immune system.

## Materials and Methods

### Oncomine and ULCAN Database

The expression levels of CLDN10 in gastric cancer were identified in the Oncomine database (https://www.oncomine.org) (Rhodes et al., 2007). In this study, a *p*-value of 0.05, a fold change of 1.5, and a gene rank in the top 10% were set as the significance thresholds. UALCAN (http://ualcan.path.uab.edu/analysis.html) (Chandrashekar et al., 2017) was used to verify the differential expression of CLDN10. This is a cancer data online analysis, mainly based on the TCGA RNA-seq and clinical data of 31 types of cancer.

#### Human Protein Atlas Database Analysis

Human Protein Atlas (HPA) is an open source database that helps researchers explore protein expression profiles in human tumor tissues and cells (https://www.proteinatlas.org/) (Uhlen et al., 2010). In this study, the protein expression levels of CLDN10 in normal and gastric cancer tissues were assessed using the database.

### GEPIA 2.0 Database

Gene Expression Profiling Interactive Analysis 2.0 (GEPIA 2.0) is a publicly accessible, online cancer database designed to analyze various cancer data based on TCGA data (http://gepia2.cancer-pku.cn/) (Tang et al., 2019). The correlation between the expression levels of CLDN10, disease-free survival (DFS) and overall survival (OS) of the patients with gastric cancer was analyzed through GEPIA 2.0. The results are shown through survival curves. In addition, the GEPIA 2.0 database was used to confirm the correlation between CLDN10 expression in gastric cancer and a number of immune cell gene markers. The correlation analysis of CLDN10 expression and signatures was determined by Spearman’s correlation with statistical significance (*p* < 0.05 was considered significant).

### Kaplan–Meier Plotter Database

Kaplan-Meier Plotter (http://kmplot.com/analysis/) (Szász et al., 2016) is an online comprehensive database that contains gene expression and clinical data for 21 types of tumors with large sample sizes for the breast (n = 6,234), ovarian (n = 2,190), lung (n = 3,452) and gastric (n = 1,440) cancer cohorts. This database was used to investigate the correlation between CDLN10 expression and the prognosis and clinicopathological features of gastric cancer through Hazard ratios (HRs) of 95%, confidence intervals (CIs), and log-rank *p*-value.

### GeneMAMIA and LinkedOmincs Database

GeneMANIA (https://genemania.org/) is an effective and reliable network analysis tool, which can help to establish protein-protein interaction (PPI) network and predict gene function (Warde-Farley et al., 2010). With this platform, we can analyze the protein interaction network, pathway and co-expression related to target genes (Mostafavi and Morris 2012). Using this tool, we created a visual PPI network of CLDN10. The LinkedOmics (http://www.linkedomics.org/) online platform can be used to analyze the multi-omics and clinical data of 33 types of cancers in TCGA database (Vasaikar et al., 2018). The interconnections, functional enrichment analysis of CLDN10 were analyzed by LinkedOmics. The rank criterion was false discovery rate (FDR) < 0.05, and 500 simulations were carried out.

### TIMER 2.0 Database

The Tumor Immune Estimation Resource 2.0 (TIMER2.0) database (Li, Fu, et al., 2020) is a publicly available immunity and gene expression repository. A variety of computational methods were undertaken (including deconvolution) to analyze and visualize the expression profile data from the TCGA to show the characteristics of tumor immune infiltration and gene expression of various cancers. Firstly, the Gene DE module was used to study the differential expression of CLDN10 in various tumors. Secondly, CLDN10 expression and its correlation with the level of immune cell infiltration in STAD were evaluated by the Gene module. Additionally, the SCNA module was used to analyze the relationship between somatic copy number alterations (SCNA) and immune infiltration level. Furthermore, the Gene_Corr module was used to verify the correlation of CLDN10 expression with gene expression markers of tumor infiltrating immune cells by Spearman analysis. The levels of gene expressin were demonstrated by log2 TPM.

### Gastric Tissue Specimens

In this study, 10 paired gastric cancer tissues and adjacent normal tissues were collected from the Eighth Affiliated Hospital of Sun Yat-sen University, Shenzhen, China. The collection and use of the samples were approved by the ethics committee of the Eighth Affiliated Hospital of Sun Yat-sen University.

### Cell Culture

The gastric cancer cell lines (AGS, NCI-N87, SNU1, KATOIII and MKN28) and human gastric mucosa cell line (GES1) were obtained from Procell life science and Technology Co., Ltd (Wuhan, China). All the cell lines were cultured in RPMI-1640 medium (GIBCO, Los Angeles, CA, United States) with 10% fetal bovine serum (FBS, Gibco) and at 37°C in a 95% air, 5% CO_2_ humidified incubator.

### RT-PCR Analysis

Human gastric cancer tissues were homogenized in Trizol (ThermoFisher Scientific) by tissue homogenizer (ThermoFisher Scientific). And the tissues and cells total RNA extracted by Trizol was used for reverse transcriptions *via* the primescript RT reagent (Takara Bio Inc., Japan) and then involved in the qPCR *via* SYBR Green PCR kit (Takara Bio Inc., Japan) and LightCycler96 Real-time PCR instrument (Roche). The analysis was conducted triply and GAPDH was used as internal reference. The following primers were used:

CLDN10 forward primer: 5′-GCA​TGT​AGA​GGA​CTT​ATG​ATC​GC-3′

CLDN10 reverse primer: 5′-TCC​GAC​TTT​GGT​ACA​CTT​CAT​TC-3′

GAPDH forward primer: 5′-GTC​TCC​TCT​GAC​TTC​AAC​AGC​G-3′

GAPDH reverse primer: 5′-ACC​ACC​CTG​TTG​CTG​TAG​CCA​A-3′

### Statistical Analysis

The data was analyzed using the GraphPad Prism (version 8.0). Student’s t-test was used to compare the difference of mRNA expression of CLDN10 between gastric cancer tissue and adjacent normal tissues. When *p* value <0.05 was considered statistically significant.

## Results

### Expression Analysis

**FIGURE 1:**
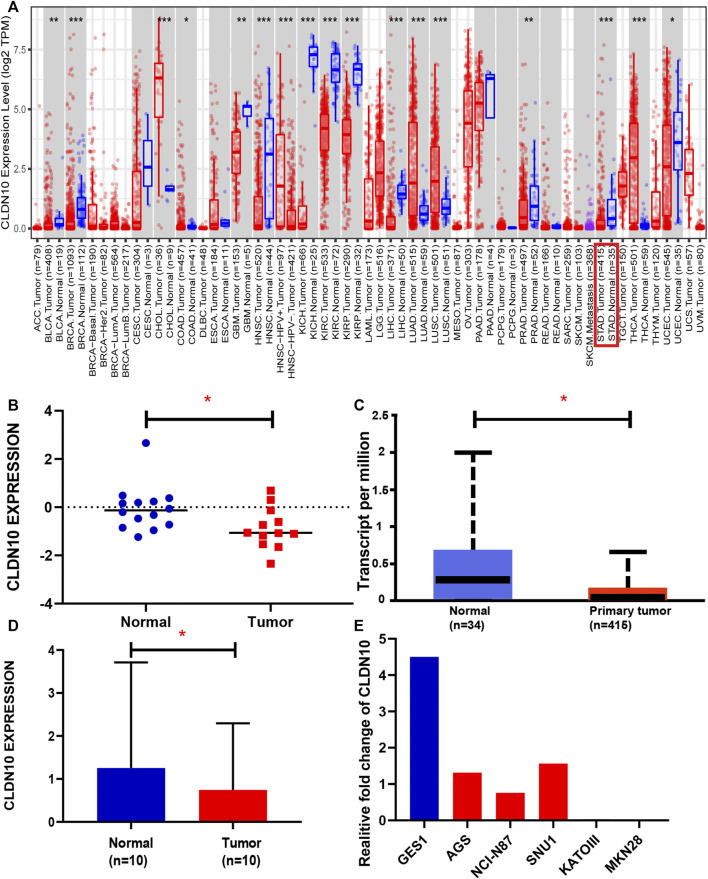
**(A)** Human CLDN10 expression levels in different tumor types from TCGA database were determined using TIMER 2.0. **(B,C)** CLDN10 expression was lower in STAD compared with normal tissues based on data in the Oncomine (THRESHOLD = 0.05, Fold change = 1.5, Data type = mRNA, *p* < 0.05) and UALCAN (*p* = 0.028). **(D,E)** Expression levels of CLDN10 in 10 pairs of gastric cancer tissues, adjacent normal tissues and different cell lines. (*p < 0.05, **p < 0.01, ***p < 0.001).

**FIGURE 2:**
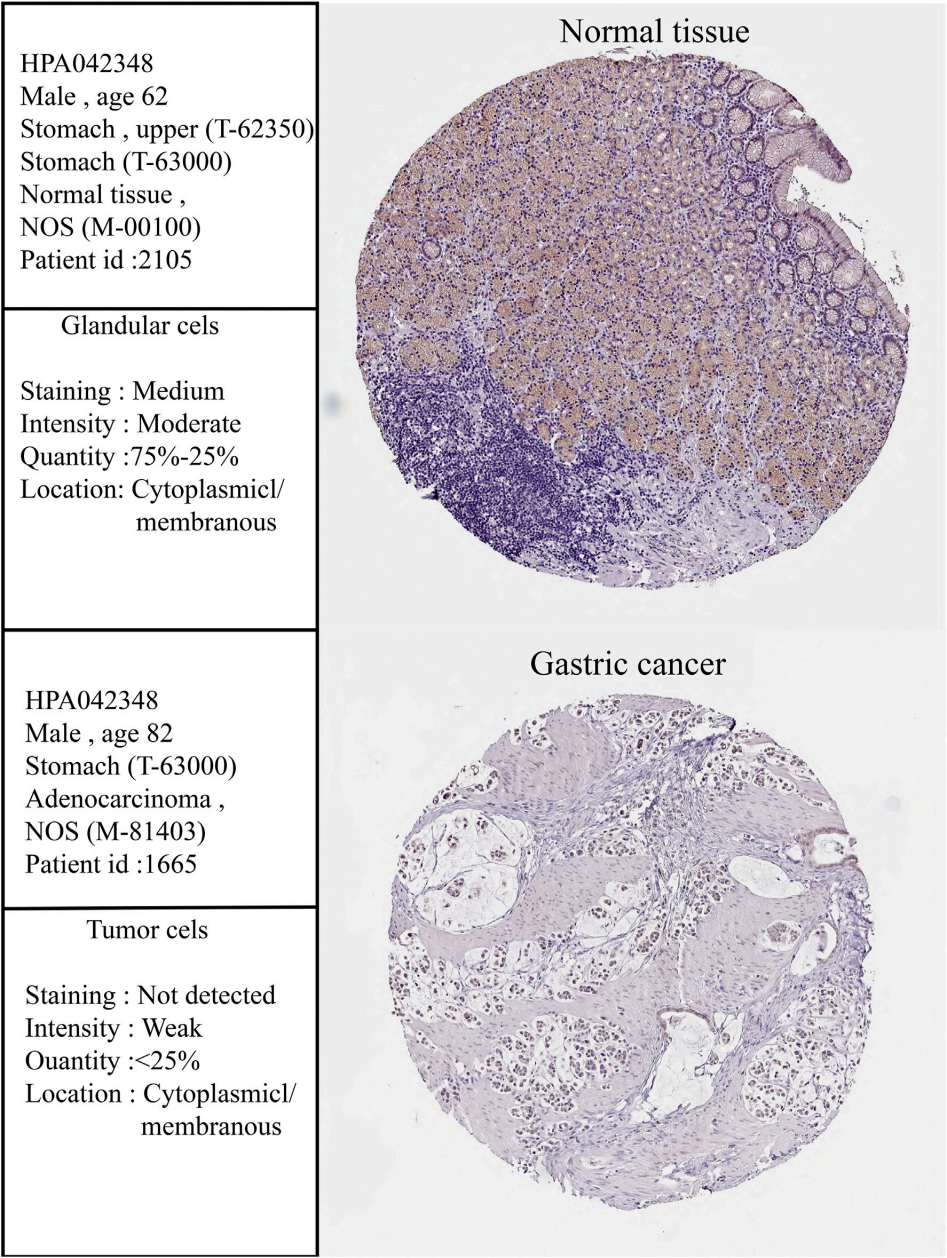
Immunohistochemistry (IHC) of CLDN10 expression in gastric cancer tissues and corresponding normal tissues based on the Human Protein Atlas (HPA).

To determine the differential expression of CLDN10 in both gastric cancers and various other cancer types, we used the TIMER 2.0 database to investigate the CLDN10 mRNA expression in human cancers. To more accurately assess CLDN10 mRNA expression in gastric cancer, we used the Oncomine and UALCAN database to verify CLDN10 expression levels. As shown in Figure 1A, CLDN10 expression was significantly down-regulated in bladder urothelial carcinoma (BLCA), breast invasive carcinoma (BRCA), colon adenocarcinoma (COAD), glioblastoma multiforme (GBM), head and neck cancer (HNSC), kidney chromophobe (KICH), kidney renal clear cell carcinoma (KIRC), kidney renal papillary cell carcinoma (KIRP), liver hepatocellular carcinoma (LIHC), prostate adenocarcinoma (PRAD), stomach adenocarcinoma (STAD) and uterine corpus endometrial carcinoma (UCEC) compared to adjacent normal tissues. However, CLDN10 expression was higher in cholangiocarcinoma (CHOL), adenocarcinoma (LUAD), lung squamous cell carcinoma (LUSC) and thyroid carcinoma (THCA) than in the adjacent normal tissues. Moreover, data from Oncomine (Figure 1B) and UALCAN (Figure 1C) confirmed that CLDN10 was down-expressed in gastric cancer. Furthermore, the immunohistochemical pictures provided by the HPA database indicated that CLDN10 was localized in the cytoplasm and membrane, and his protein expression level in gastric cancer was also lower than that in normal tissues (Figure 2). Lastly, we detected the mRNA expression level of 10 paired GC tissues and adjacent normal tissues and GC cell lines by Real-Time PCR to verify the down-regulation of CLDN10 in gastric cancer. The results demonstrated that mRNA expression level of CLDN10 was reduced when compared with adjacent normal tissues (Figure 1D). And CLDN10 was also down-regulated in gastric cancer cell lines when compared with human gastric mucosa cell line (Figure 1E).

### Prognostic and Clinicopathological Significance of CLDN10 Expression in Gastric Cancer

**FIGURE 3:**
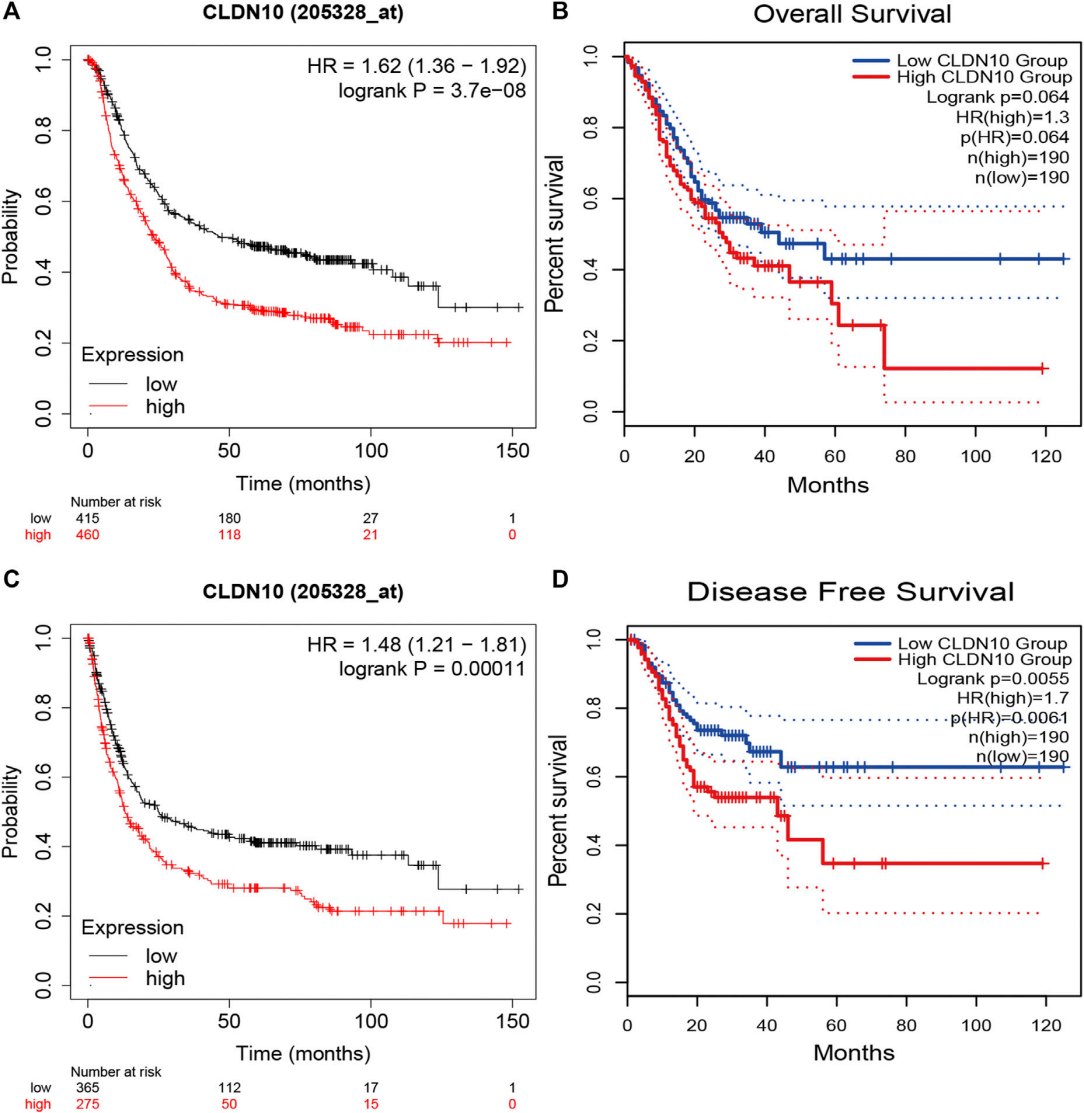
Survival analysis of CLDN10 expression in patients with gastric cancer. **(A,B)** Decreased expression of CLDN10 was associated with favorable prognosis of overall survival (Kaplan-Meier Plotter and GEPIA2.0), **(C)** progression-free survival (Kaplan-Meier Plotter) and **(D)** Disease-free survival (GEPIA2.0).

In order to better understand potential correlations of CLDN10 expression in gastric cancer, we used Kaplan-Meier Plotter and GEPIA2.0 database to investigate the prognosis and clinicopathological significance of CLDN10. From the Kaplan-Meier Plotter, the results showed that overexpression of CLDN10 corresponded with an unfavorable prognosis for gastric cancer patients (Figure 3A, OS HR = 1.62, 95% CI = 1.36–1.92, *p* = 3.7e-8; Figure 3C, PFS HR = 1.48, 95% CI = 1.21–1.81, *p* = 0.00011). Moreover, we used GEPIA2.0 to verify this association and got similar results (Figure 3B, OS HR = 1.3, *p* = 0.064; Figure 3D, DFS HR = 1.7, *p* = 0.0055).

To further understand the in-depth significance and internal mechanism of the differential expression of CLDN10 in gastric cancer tissues and adjacent normal tissues, we explored the interrelation between CLDN10 expression levels and the clinicopathological characteristics of gastric cancer using the Kaplan-Meier Plotter database. High CLDN10 expression was associated with poor OS and PFS in both male and female gastric cancer patients (Table 1 **;** *p* < 0.05). TNM staging is a widely accepted standard for cancer classification, which can predict the prognosis of gastric cancer. It means the invasive depth of primary tumor (T), regional lymph node (N), and distant metastasis (M) (Edge and Compton 2010; Cuccurullo and Mansi 2011). Regardless of the clinical stage, high CLDN10 expression was related to poorer OS, but only stage 2 was related to PFS. The expression of CLDN10 in T2 gastric cancer patients was associated with OS and PFS (OS *p* = 0.03; PFS *p* = 0.027), and the value of HR has a decreasing trend with increasing tumor development. A statistically significant correlation between CLDN10 expression and gastric cancer prognosis was observed in stage N0\N1\N3\N1+2 + 3 patients but not N2. The expression of CLDN10 was statistically correlated with OS and PFS in stage M0 gastric cancer, while only OS in stage M1. According to the Lauren classification, it was observed that the expression of CLDN10 in patients with intestinal-type gastric cancer was related to OS and PFS. In mixed-type group, it was associated with OS but not with PFS, while in diffuse-type gastric cancer, no statistically significant association was observed. In terms of treatment, the expression of CLDN10 in gastric cancer patients who have only undergone surgery was related to OS and PFS (OS *p* = 0.017; PFS *p* = 0.044). However, it was not significantly related to the prognosis of patients who had received 5-FU-based adjuvant chemotherapy. In addition, CLDN10 expression was associated with OS and PFS in HER2-negative gastric cancer patients (OS *p* = 2.1e-07; PFS *p* = 0.00033), but not with HER2-positive patients.

**TABLE 1:** Correlations of CLDN10 expression and clinicopathological significance in gastric cancer.

	OS PFS			PFS		
	N	HR	*p* value	N	HR	*p* Value
Sex
Female	236	2.18(1.46–3.28)	0.0001	201	1.98(1.31–2.99)	0.00095
Male	544	1.64(1.32–2.04)	5.3544e-6	437	1.61(1.21–2.15)	0.00092
Stage
1	67	3.22(1.11–9.32)	0.023	60	2.37(0.77–7.3)	0.12
2	140	2.55(1.33–4.88)	0.0034	131	2.42(1.32–4.42)	0.0031
3	305	1.7(1.27–2.27)	3e-04	186	0.8(0.53–1.23)	0.31
4	148	1.66(1.07–2.59)	0.023	141	1.54(0.96–2.46)	0.073
Stage T
2	241	1.56(1.02–2.39)	0.037	239	1.59(1.05–2.41)	0.027
3	204	0.83(0.58–1.19)	0.31	204	0.77(0.55–1.1)	0.15
4	38	0.58(0.23–1.5)	0.26	39	0.61(0.24–1.51)	0.28
Stage N
0	74	3.06(1.25–7.48)	0.01	72	2.96(1.21–7.22)	0.013
1	225	2.01(1.26–3.2)	0.0028	222	1.84(1.18–2.88)	0.0063
2	121	0.88(0.54–1.43)	0.6	125	0.74(0.48–1.13)	0.16
3	76	1.82(1.03–3.22)	0.036	76	1.92(1.05–3.52)	0.032
1 + 2+3	422	1.33(1.02–1.74)	0.034	423	1.28(0.99–1.65)	0.059
Lauren classification
intestinal	320	2.15(1.55–2.98)	2.3e-06	263	1.6(1.13–2.27)	0.0079
diffuse	241	1.28(0.89–1.83)	0.1	231	1.18(0.82–1.69)	0.37
mixed	32	1.71(0.54–5.38)	0.36	28	0.43(0.15–1.21)	0.099
Treatment
surgery alone	380	1.54(1.08–2.19)	0.017	375	1.43(1.01–2.02)	0.044
5 FU based adjuvant	152	0.74(0.5–1.08)	0.12	152	0.73(0.49–1.08)	0.12
other adjuvant	86	0.37(0.15–0.89)	0.021	80	0.42(0.19–0.93)	0.028
HER2
negative	532	1.8(1.44–2.26)	2.1e-07	408	1.62(1.24–2.12)	0.00033
positive	343	1.25(0.95–1.64)	0.11	232	1.31(0.9–1.92)	0.16
Stage M
0	444	1.42(1.07–1.87)	0.013	443	1.35(1.03–1.76)	0.027
1	56	2(1.08–3.71)	0.024	56	1.82(0.97–3.39)	0.058

OS, overall survival; PFS, Progression-free survival, HR, Hazard ratio.

### Protein-Protein Interaction Network and Function Enrichment Analysis of CLDN10

**FIGURE 4:**
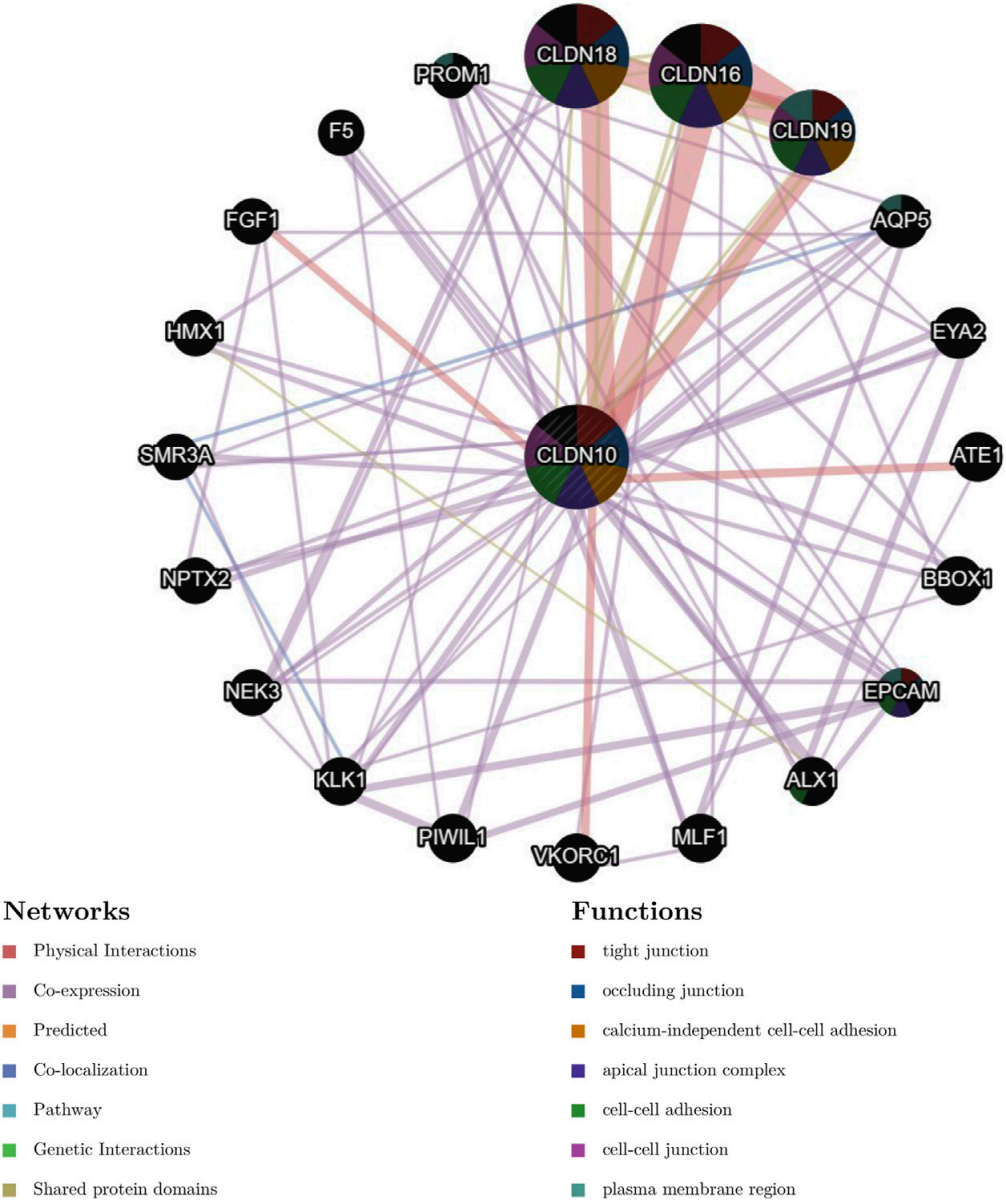
Protein-protein interaction network of associated genes generated by GENEMANIA.

To predict the potential interactions between CLDN10 and other cancer-related proteins, we constructed a PPI network using a GeneMANIA database. The results showed that CLDN16, CLDN18 and CLDN19 were the proteins most closely related to CLDN10, mainly involved in tight junction, occuluding junction, calcium-independent cell-cell adhesion and apical junction complex (Figure 4 **)**.

**FIGURE 5:**
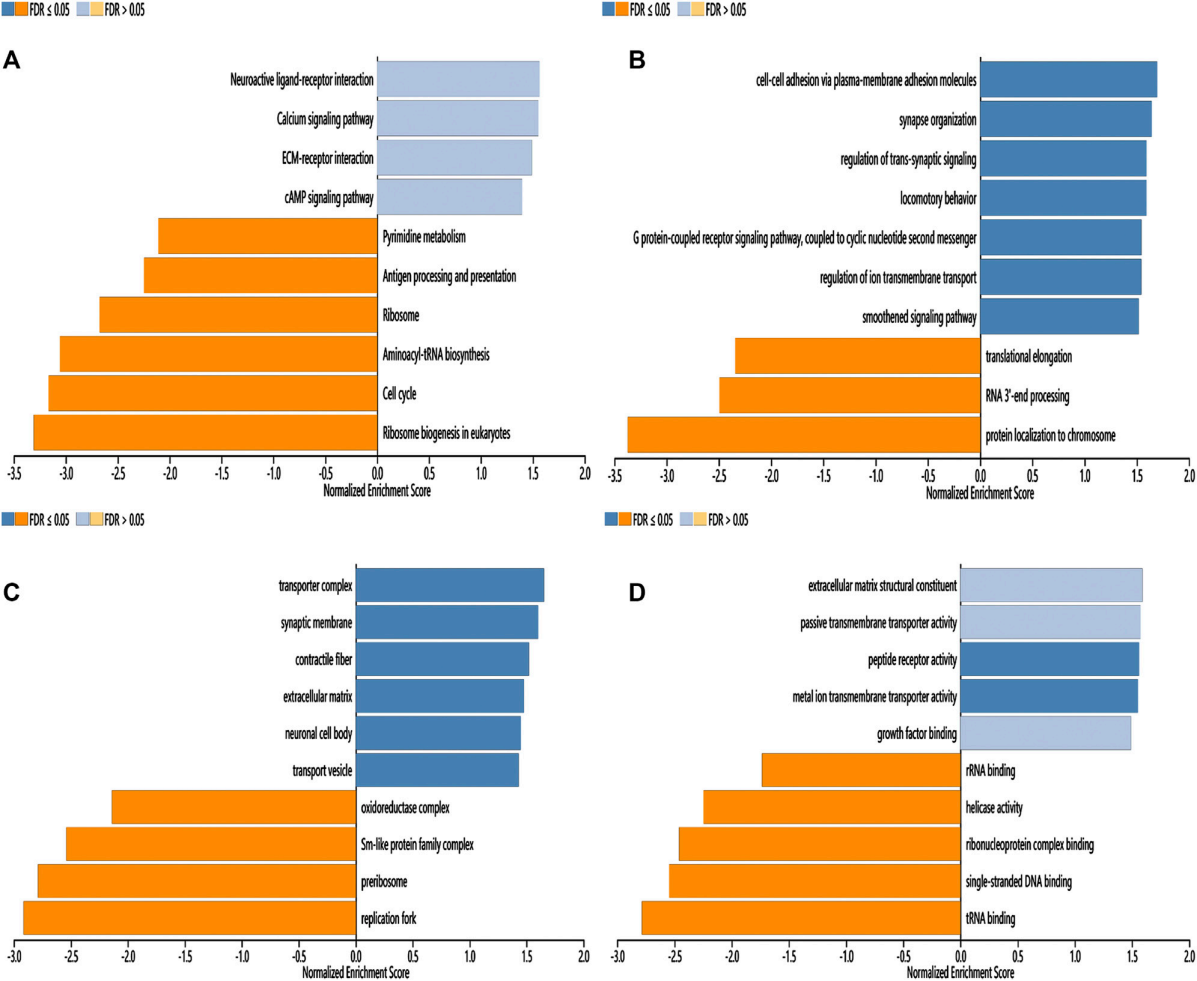
Function enrichment analysis of CLDN10 in gastric cancer (LinkedOmics). **(A)** KEGG pathways analysis. **(B)** Biological process analysis. **(C)** Cellular component analysis. **(D)** Molecular function analysis.

To further investigate the possible molecular mechanism of CLDN10 in gastric cancer, the LinkInterpreter module of LinkedOmics were utilized to analyse function enrichment. The KEGG pathway indicated that CLDN10 and its related genes were primarily associated with Neuroactive ligand-receptor interaction, Calcium signaling pathway, ECM-receptor interaction, cAMP signaling pathway, Aminoacyl-tRNA biosynthesis, Cell cycle and Ribosome biogenesis in eukaryotes (Figure 5A). GO_BP (biological process) was mainly related to cell-cell adhesion *via* plasma-membrane adhesion molecules, synapse organization, regulation of *trans*-synaptic signaling, protein localization to chromosome and tRNA metabolic process (Figure 5B). GO_CC (cell component) was mainly expressed in transporter complex, synaptic membrane and oxidoreductase complex (Figure 5C). GO_MF (molecular function) was mainly associated with extracellular matrix structural constituent, passive transmembrane transporter activity, metal ion transmembrane transporter activity, ribonucleoprotein complex binding and structural constituent of ribosome (Figure 5D). In conclusion, CLDN10 might be involved in signal transmission, transmembrane transport and metabolism.

### CLDN10 Expression and Somatic Copy Number Alternations Were Correlated With Immune Cell Infiltration in Gastric Cancer

**FIGURE 6:**
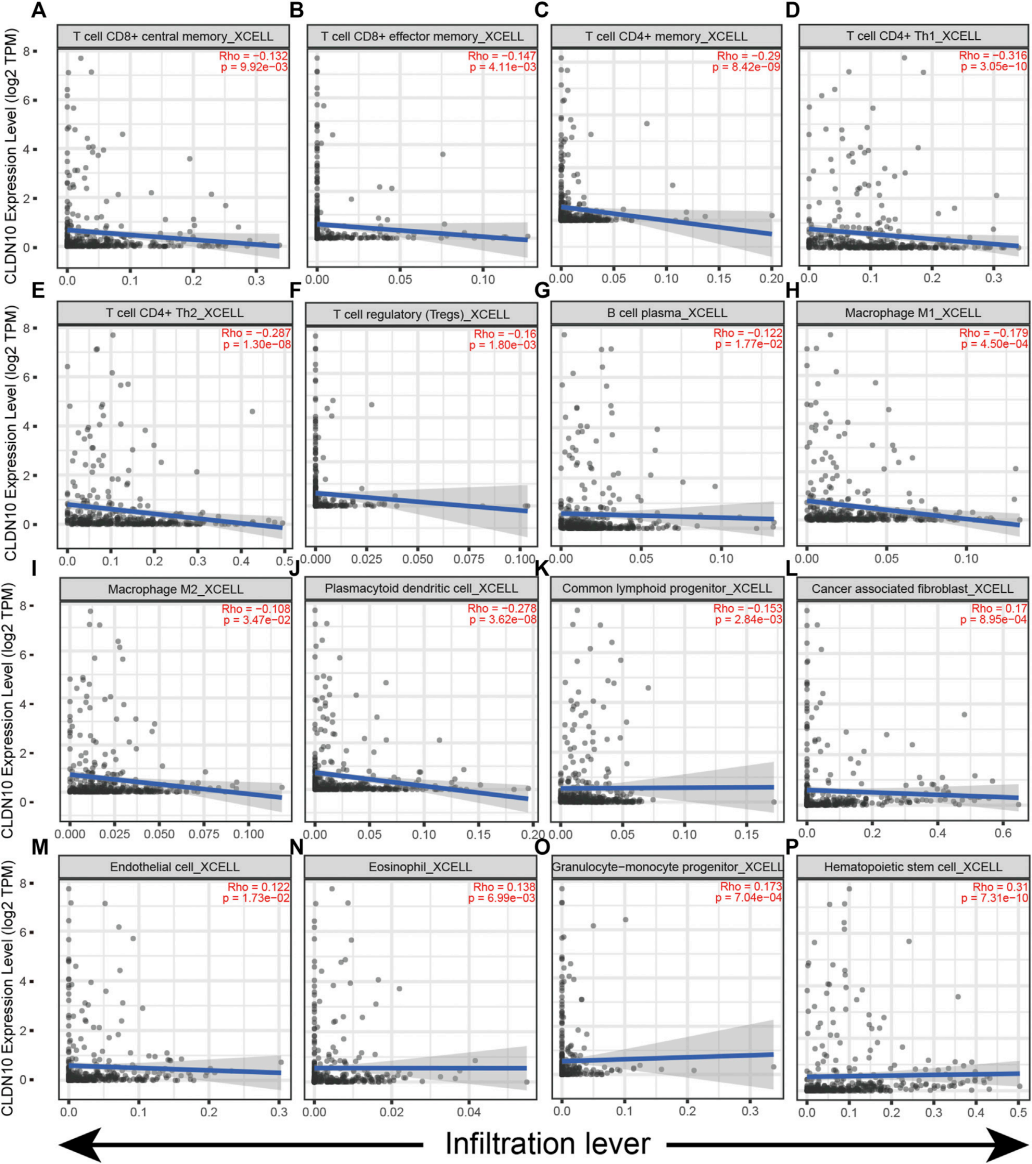
Correlation of CLDN10 expression with immune infiltration level in STAD. **(A–K)** The expression of CLDN10 was negatively related to the infiltrating levels of these immune cells (T cell CD8^+^ central memory, T cell CD8^+^ effector memory, T cell CD4^+^ memory, T cell CD4^+^ Th1, T cell CD4^+^ Th2, T cell regulatory (Tregs), B cell plasma, Macrophage M1, Macrophage M2, Plasmacytoid dendritic cell, and Common lymphoid progenitor) in STAD. **(L–P)** CLDN10 expression was positively related to the infiltrating levels of Cancer associated fibroblast, Endothelial cell, Eosinophil, Granulocyte−monocyte progenitor and Hematopoietic stem cell in STAD.

**FIGURE 7:**
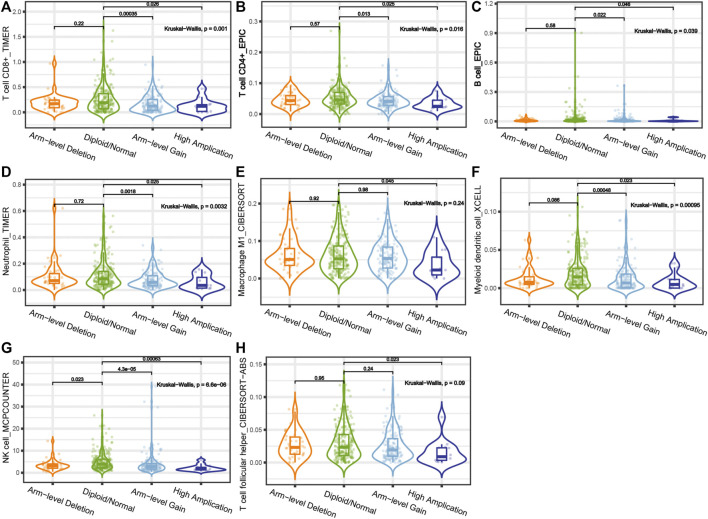
Correlations between somatic copy number variation and immune infiltration levels of **(A)** CD8+ T cells, **(B)** CD4+ T cells, **(C)** B cells, **(D)** neutrophil, **(E)** macrophages M1, **(F)** myeloid dendritic cells, **(G)** NK cells and **(H)** T cell follicular helper in STAD.

The gene module in the TIMER 2.0 database was used to investigate the relationship between CLDN10 expression and immune cell infiltration in STAD (Figure 6). It was seen that CLDN10 expression level was negatively related to the infiltrating levels of T cell CD8^+^ central memory, T cell CD8^+^ effector memory, T cell CD4^+^ memory, T cell CD4^+^ Th1, T cell CD4^+^ Th2, T cell regulatory (Tregs), B cell plasma, Macrophage M1, Macrophage M2, Plasmacytoid dendritic cell, and Common lymphoid progenitor in STAD, while positively correlated with Cancer associated fibroblast (CAF), Endothelial cell, Eosinophil, Granulocyte−monocyte progenitor and Hematopoietic stem cell (*p* < 0.05). Most of the immune cells mentioned above were negatively associated with CLDN10 level, which meant that there were more infiltrating immune cells in the tumor microenvironment in gastric cancer patients with low expression of CLDN10. This could partly explain why a decline in CLDN10 was associated with better outcomes. Moreover, we analyzed and compared the immune infiltration levels among gastric cancer patients with the presence of different SCNA status for CLDN10 (Figure 7). It was observed that high amplification in STAD was negatively associated with the infiltration of CD8^+^ T cells, CD4^+^ T cells, B cells, neutrophil, macrophages M1, myeloid dendritic cells, NK cells and T cell follicular helper (*p* < 0.05). Furthermore, arm-level gain was also inversely correlated with the above immune cell infiltration (*p* < 0.05), except for macrophage M1 and T cell follicular helper. However, only the level of NK cell infiltration was significantly correlated with the arm-level deletion (*p* < 0.05). These results showed us that SCNA of CLDN10 may affect the level of immune cell infiltration.

### Correlation Analyses Between Immune Marker Genes and CLDN10 Expression

The TIMER 2.0 database interrogation concentrated on the correlations between CLDN10 in STAD and related marker genes of infiltrating immune cells (Table 2). After purity-related adjustments, it was shown that CLDN10 levels were notably related to some marker genes in a variety of immune cells, including T cells (general), M1 Macrophages, Neutrophils, Natural killer cells, Dendritic cells (DCs) and different functional T cells, such as Tregs, Th1, Tfh, Th17 and exhausted T cells. It was of note that the levels of the main markers of B cell (CD19, CD79A), Tfh (BCL6, IL21) and exhausted T cells (PD1, CTLA4, LAG3, TIM3, GZMB) were all significantly correlated with CLDN10 levels in STAD. Tfh could promote the differentiation of B cells into memory B cells and the survival of plasma cells by providing signals to B cells, which played an important role in humoral immunity (Ise et al., 2014; Cao et al., 2021). In exhausted T cells, these gene markers (PD1, CTLA4, LAG3, TIM3 and GZMB) were consistently significantly correlated with the expression of CLDN10, which further suggested that the expression level of CLDN10 in gastric cancer is related to immune infiltration.

**TABLE 2:** Correlation analysis between CLDN10 and relate genes and markers of immune cells in TIMER2.0.

Description	Gene markers	STAD			
		None		Purity	
		Cor	*P*	Cor	*P*
CD8 + T cell	CD8A	−0.063	0.198	−0.077	0.133
	CD8B	0.054	0.274	0.058	0.262
T cell (general)	CD3D	−0.083	0.0899	−0.116	**0.0245**
	CD3E	−0.045	0.356	−0.079	0.126
	CD2	−0.066	0.18	−0.091	0.0781
B cell	CD19	0.203	**3.21E-05**	0.174	**6.80E-04**
	CD79A	0.153	**1.76E-03**	0.127	**1.34E-02**
Monocyte	CD86	−0.059	2.32E-01	−0.081	0.115
	CSF1R	0.027	0.589	−0.003	0.954
TAM	CCL2	0.106	**0.0305**	0.098	0.0569
	CD68	−0.065	0.19	−0.068	0.189
	IL10	0.049	0.32	0.02	0.698
M1 Macrophage	NOS2	−0.169	**5.54E-04**	−0.19	**2.04E-04**
	IRF5	0.083	9.18E-02	0.072	0.162
	PTGS2	0.111	**2.34E-02**	0.099	5.40E-02
M2 Macrophage	CD163	−0.032	0.519	−0.047	0.362
	VSIG4	−0.01	0.843	−0.015	0.768
	MS4A4A	0.007	0.887	−0.007	0.888
Neutrophils	CEACAM8	0.08	0.106	0.078	0.127
	ITGAM	0.019	0.695	0.001	0.214
	CCR7	0.146	**2.93E-03**	0.12	**1.97E-02**
Natural killer cell	KIR2DL1	0.019	0.698	0.013	0.795
	KIR2DL3	−0.052	0.289	−0.063	0.224
	KIR2DL4	−0.129	**8.65E-03**	−0.132	**1.01E-02**
	KIR3DL1	−0.047	0.344	−0.048	0.349
	KIR3DL2	−0.062	0.206	−0.071	0.17
	KIR3DL3	−0.053	0.278	−0.056	0.274
	KIR2DS4	−0.087	7.65E-02	−0.094	6.71E-02
Dendritic cell	HLA-DPB1	−0.038	0.437	−0.064	0.21
	HLA-DQB1	−0.079	0.11	−0.103	**4.49E-02**
	HLA-DRA	−0.08	0.104	−0.105	**4.05E-02**
	HLA-DPA1	−0.052	0.29	−0.076	0.141
	CD1C	0.238	**9.35E-07**	0.219	**1.65E-05**
Th1	NRP1	0.124	**1.17E-02**	0.102	**4.66E-02**
	ITGAX	−0.008	0.878	−0.031	0.547
	TBX21	−0.056	0.256	−0.085	9.76E-02
	STAT1	−0.13	**7.81E-03**	−0.139	**6.69E-03**
	IFNG	−0.229	**2.47E-06**	−0.243	**1.64E-06**
	TNF	0.021	0.677	−0.01	0.845
Th2	GATA3	0.047	0.34	0.032	0.528
	STAT6	0.023	0.633	0.036	0.479
	STAT5A	−0.003	0.947	−0.022	0.67
	IL13	−0.037	0.455	−0.058	0.262
Tfh	BCL6	0.299	**4.83E-10**	0.297	**3.49E-09**
	IL21	−0.09	6.64E-02	−0.109	**3.34E-02**
Th17	STAT3	0.138	**5.01E-03**	0.131	**1.05E-02**
	IL17A	−0.052	0.292	−0.064	0.215
Treg	FOXP3	−0.11	**2.51E-02**	−0.132	**1.03E-02**
	CCR8	−0.035	0.478	−0.057	0.269
	STAT5B	0.178	**2.73E-04**	0.153	**2.78E-03**
	TGFB1	0.104	**3.33E-02**	0.08	0.121
T cell exhaustion	PD1(PDCD1)	−0.088	7.17E-02	−0.102	**4.62E-02**
	CTLA4	−0.106	**3.02E-02**	−0.122	**1.74E-02**
	LAG3	−0.151	**2.09E-03**	−0.161	**1.69E-03**
	TIM3(HAVCR2)	−0.093	5.84E-02	−0.109	**3.37E-02**
	GZMB	-−0.228	**2.57E-06**	−0.244	**1.57E-06**

STAD, Stomach adenocarcinoma; TAM, tumor-associated macrophage; Cor, R value of Spearman’s correlation; Purity, correlation adjusted by purity.

## Discussion

It has been reported that abnormal claudin expression relates to the occurrence, development and metastasis of malignant tumors. This has potential clinical value in the diagnosis and treatment of tumors and could therefore be used as a diagnostic marker or a target for immunotherapy (Swisshelm, Macek, and Kubbies 2005; Kominsky 2006; Tabariès and Siegel 2017). The low expression of CLDN1 in lung adenocarcinoma promoted the migration, invasion and metastasis of cancer cells, and was associated with a shorter OS (Chao et al., 2009). CLDN3 and CLDN4 is overexpressed in ovarian cancer and increases cell invasion and motility, thereby promoting tumor occurrence and metastasis (Agarwal, D'Souza, and Morin 2005). The low expression of CLDN18 in gastric cancer could be used as a marker of adverse outcome (Sanada et al., 2006). Furthermore, anti-claudin 18.2 antibody has been used in clinical trials to treat advanced gastric cancer, showing the broad prospects of claudins in tumor treatment (Singh, Toom, and Huang 2017; Sahin et al., 2021). However, to date, the clinical significance of CLDN10 expression in gastric cancer has remained unclear.

In this study, Oncomine, UALCAN, HPA and TIMER2.0 databases were used to evaluate the expression levels of CLDN10 mRNA and protein in gastric cancer. The results from these four databases mutually confirmed that the expression level of CLDN10 in gastric cancer tissues was lower than that in adjacent normal tissues. More interestingly, according to the information provided by HPA, CLDN10 protein was located in cytoplasm and cell membrane in both gastric cancer and normal tissues. This feature was consistent with the function of CLDN10 in regulating substance transmembrane transport. In addition, we analyzed and verified the expression level of CLDN10 in gastric cancer samples and cell lines, and the results were consistent with the online database, further confirming the down-regulation role of CLDN10 in gastric cancer.

Next, we evaluated the relationship between CLDN10 expression level and prognosis of gastric cancer. Generally speaking, abnormal expression of CLDN10 could affect the structure of tight junction, resulting in loss of cell adhesion, thereby promoting metastasis of tumor cells, enhancing tumor invasiveness and leading to poor prognosis (Martin and Jiang 2009). The epithelial/mesenchymal transition (EMT) theory can partially explain this process (Chaffer and Weinberg 2011). Incredibly, this paper drew the opposite conclusion. It was seen that low CLDN10 expression was associated with better OS, PFS, and DFS in gastric cancer patients. To explain this anomaly, we conducted a series of analyses to find the answer.

Firstly, the relationship between CLDN10 and various clinicopathological features was assessed. It was found that the high expression of CLDN10 had the highest HR value in stage 1, stage T2, stage N0, intestinal type, surgery only and HER2-negative subgroups, and the correlation was statistically significant. With regard to lymph node metastasis, we found that the expression of CLDN10 was statistically correlated with OS and PFS in all stage except stage N2, suggesting that the abnormal expression of CLDN10 may be related to lymph node metastasis of gastric cancer. A previous study obtained similar results to support our results (Xiang et al., 2020). This study demonstrated that abnormal CLDN10 expression was associated with lymph node metastasis in papillary thyroid carcinoma, yet patients have a better prognosis. The authors suggested that although lymph node metastasis is an adverse prognostic factor, there are other protective factors that outweigh the adverse effects of lymph node metastasis. Further analysis found a positive correlation between levels of immune-infiltrating cells in thyroid papillary carcinoma with high expression of CLDN10, thus explaining the improved prognosis.

Inspired by this study, we continued GSEA, PPI network, somatic copy number alternations and immune correlation analysis. Based on the results obtained, we speculated that CLDN10 was mainly involved in signal transmission, transmembrane transport and metabolism. Therefore, when CLDN10 was abnormally expressed, these functions could be impaired leading to the occurrence and migration of tumors.

Moreover, our results showed that the decreased expression of CLDN10 was associated with increased infiltration of several major immune cells, potentially enhancing the body’s anti-tumor immune response, which partly explains why low expression of CLDN10 was associated with better prognosis in gastric cancer. This result was similar to previous study, demonstrating that increased levels of immune-infiltrating cells may improve the survival of tumors, and this effect is greater than the adverse effect of lymph node metastasis (Xiang et al., 2020). Somatic copy number alterations are one of the driving factors for tumorigenesis and have been used in tumor prognosis prediction and treatment (Stuart and Sellers 2009; Hieronymus et al., 2018). The relationship between the level of immune cell infiltration and SCNA was then assessed. It could be found from the violin diagram that the SCNA types of high amplification and arm-level gain mainly affected the level of immune infiltration (Figure 6). High amplification of CLDN10 correlated with a decrease in the infiltration level of some immune cells and the same situation occurred in the arm-level gain group, with the exception of macrophage M1 and T cell follicular helper. In addition, arm-level deletion was only found to be statistically associated with the changes in NK cell infiltration level. These results shed some light on the potential influence of SCNA status on the level of immune cell infiltration.

Furthermore, the relationship between CLDN10 expression and gene markers of immune cells was analyzed. The expression level of CLDN10 was seen to have a statistically significant positive or negative correlation with the gene markers of many immune cells (Table 2). Tfh could promote B cell differentiation, antibody class switching and germinal center formation (Jogdand, Mohanty, and Devadas 2016). Our results showed that CLDN10 expression significantly correlated to the expression of B cells (CD19, CD79A) and Tfh cells (BCL6, IL21) markers, which indicated a potential mechanism for CLDN10 to regulate B cell function in gastric cancer. In addition, PD1, CTLA4, LAG3, TIM3 and GZMB were common inhibitory receptors (IRs) in the tumor microenvironment, which could inhibit immune responses and even cause immune escape or tolerance after binding with corresponding ligands. Immune checkpoint inhibitors that targeted and antagonized these IRs have been developed to treat a variety of tumors with remarkable success and are promising therapies for the future (Kato et al., 2019; André et al., 2020; Jabbour et al., 2020; van Dijk et al., 2020). The results shown here demonstrate that CLDN10 expression negatively correlates to these gene markers. These correlations suggest that CLDN10 might participate in immune escape by regulating T cell exhaustion. Furthermore, significant correlations could be found between CLDN10 expression and the regulation of some markers of T cell (CD3D), M1 Macrophage (NOS2), Neutrophils (CCR7), Natural killer cell (KIR2DL4), Dendritic cell (HLA-DQB1, HLA-DRA and CD1C), Th1 (NRP1, STAT1 and IFGN), Th17 (STAT3), Treg (FOXP3 and STAT5B) in gastric cancer. Based on the above results, CLDN10 might play an important role in the regulation of immune cell infiltration in gastric cancer.

In summary, CLDN10 is down-regulated in gastric cancer and associated with a better prognosis. Somatic copy number alternations of CLDN10 may affect the level of immune cell infiltration. And the protective effect of elevated levels of immune-infiltrating cells was greater than the adverse effect of lymph node metastasis, which ultimately presented as anticancer effect, leading to improved prognosis of gastric cancer patients.

However, these are limitations in this research. i. Due to lack of available data, the relationship between differential expression of CLDN10 and lymph node metastasis was not explored in detail; ii. further experiments are needed to verify the mechanism of CLDN10 regulating immune infiltration in the gastric cancer tumor microenvironment; iii. The regulatory mechanism of differential expression of CLDN10 and prognosis of gastric cancer needs to be further verified by well-designed studies.

## Conclusion

Down-regulated CLDN10 was associated with better overall survival (OS) in gastric cancer. And CLDN10 may serve as a potential prognostic biomarker and correlate to immune infiltration levels in gastric cancer.

## Data Availability

The datasets used and/or analyzed during the current study are available from the corresponding author upon reasonable request.

## References

[B1] Agarwal R. D'Souza T. Morin P. J. (2005). Claudin-3 and Claudin-4 Expression in Ovarian Epithelial Cells Enhances Invasion and Is Associated with Increased Matrix Metalloproteinase-2 Activity. Cancer Res. 65 (16), 7378–7385. 10.1158/0008-5472.Can-05-1036 16103090

[B2] André T. Shiu K.-K. Kim T. W. Jensen B. V. Jensen L. H. Punt C. (2020). Pembrolizumab in Microsatellite-Instability-High Advanced Colorectal Cancer. N. Engl. J. Med. 383 (23), 2207–2218. 10.1056/NEJMoa2017699 33264544

[B3] Bray F. Ferlay J. Soerjomataram I. Siegel R. L. Torre L. A. Jemal A. (2018). Global Cancer Statistics 2018: GLOBOCAN Estimates of Incidence and Mortality Worldwide for 36 Cancers in 185 Countries. CA: A Cancer J. Clinicians 68 (6), 394–424. 10.3322/caac.21492 30207593

[B4] Cao Y. Dong L. He Y. Hu X. Hou Y. Dong Y. (2021). The Direct and Indirect Regulation of Follicular T Helper Cell Differentiation in Inflammation and Cancer. J. Cel Physiol 236, 5466–5481. 10.1002/jcp.30263 33421124

[B5] Chaffer C. L. Weinberg R. A. (2011). A Perspective on Cancer Cell Metastasis. Science 331 (6024), 1559–1564. 10.1126/science.1203543 21436443

[B6] Chandrashekar D. S. Bashel B. Balasubramanya S. A. H. Creighton C. J. Ponce-Rodriguez I. Chakravarthi B. V. S. K. (2017). UALCAN: A Portal for Facilitating Tumor Subgroup Gene Expression and Survival Analyses. Neoplasia 19 (8), 649–658. 10.1016/j.neo.2017.05.002 28732212 PMC5516091

[B7] Chao Y.-C. Pan S.-H. Yang S.-C. Yu S.-L. Che T.-F. Lin C.-W. (2009). Claudin-1 Is a Metastasis Suppressor and Correlates with Clinical Outcome in Lung Adenocarcinoma. Am. J. Respir. Crit. Care Med. 179 (2), 123–133. 10.1164/rccm.200803-456OC 18787218

[B8] Cuccurullo V. Mansi L. (2011). AJCC Cancer Staging Handbook: from the AJCC Cancer Staging Manual (7th Edition). Eur. J. Nucl. Med. Mol. Imaging 38 (2), 408. 10.1007/s00259-010-1693-9

[B9] Edge S. B. Compton C. C. (2010). The American Joint Committee on Cancer: the 7th Edition of the AJCC Cancer Staging Manual and the Future of TNM. Ann. Surg. Oncol. 17 (6), 1471–1474. 10.1245/s10434-010-0985-4 20180029

[B10] Hellmann M. D. Paz-Ares L. Bernabe Caro R. Zurawski B. Kim S.-W. Carcereny Costa E. (2019). Nivolumab Plus Ipilimumab in Advanced Non-small-cell Lung Cancer. N. Engl. J. Med. 381 (21), 2020–2031. 10.1056/NEJMoa1910231 31562796

[B11] Hieronymus H. Murali R. Tin A. Yadav K. Abida W. Moller H. (2018). Tumor Copy Number Alteration burden Is a Pan-Cancer Prognostic Factor Associated with Recurrence and Death. Elife 7. 10.7554/eLife.37294 PMC6145837 30178746

[B12] Hirohashi S. Kanai Y. (2003). Cell Adhesion System and Human Cancer Morphogenesis. Cancer Sci. 94 (7), 575–581. 10.1111/j.1349-7006.2003.tb01485.x 12841864 PMC11160151

[B13] Huang G. W. Ding X. Chen S. L. Zeng L. (2011). Expression of Claudin 10 Protein in Hepatocellular Carcinoma: Impact on Survival. J. Cancer Res. Clin. Oncol. 137 (8), 1213–1218. 10.1007/s00432-011-0987-z 21647678 PMC11828256

[B14] Ise W. Inoue T. McLachlan J. B. Kometani K. Kubo M. Okada T. (2014). Memory B Cells Contribute to Rapid Bcl6 Expression by Memory Follicular Helper T Cells. Proc. Natl. Acad. Sci. 111 (32), 11792–11797. 10.1073/pnas.1404671111 25071203 PMC4136626

[B15] Jabbour S. K. Berman A. T. Decker R. H. Lin Y. Feigenberg S. J. Gettinger S. N. (2020). Phase 1 Trial of Pembrolizumab Administered Concurrently with Chemoradiotherapy for Locally Advanced Non-small Cell Lung Cancer. JAMA Oncol. 6 (6), 848–855. 10.1001/jamaoncol.2019.6731 32077891 PMC7042914

[B16] Janjigian Y. Y. Bendell J. Calvo E. Kim J. W. Ascierto P. A. Sharma P. (2018). CheckMate-032 Study: Efficacy and Safety of Nivolumab and Nivolumab Plus Ipilimumab in Patients with Metastatic Esophagogastric Cancer. Jco 36 (28), 2836–2844. 10.1200/jco.2017.76.6212 PMC6161834 30110194

[B17] Jogdand G. M. Mohanty S. Devadas S. (2016). Regulators of Tfh Cell Differentiation. Front. Immunol. 7, 520. 10.3389/fimmu.2016.00520 27933060 PMC5120123

[B18] Kang Y.-K. Boku N. Satoh T. Ryu M.-H. Chao Y. Kato K. (2017). Nivolumab in Patients with Advanced Gastric or Gastro-Oesophageal junction Cancer Refractory to, or Intolerant of, at Least Two Previous Chemotherapy Regimens (ONO-4538-12, ATTRACTION-2): a Randomised, Double-Blind, Placebo-Controlled, Phase 3 Trial. The Lancet 390 (10111), 2461–2471. 10.1016/s0140-6736(17)31827-5 28993052

[B19] Kato K. Cho B. C. Takahashi M. Okada M. Lin C.-Y. Chin K. (2019). Nivolumab versus Chemotherapy in Patients with Advanced Oesophageal Squamous Cell Carcinoma Refractory or Intolerant to Previous Chemotherapy (ATTRACTION-3): a Multicentre, Randomised, Open-Label, Phase 3 Trial. Lancet Oncol. 20 (11), 1506–1517. 10.1016/s1470-2045(19)30626-6 31582355

[B20] Kominsky S. L. (2006). Claudins: Emerging Targets for Cancer Therapy. Erm 8 (18), 1–11. 10.1017/s1462399406000056 16887048

[B21] Krause G. Winkler L. Mueller S. L. Haseloff R. F. Piontek J. Blasig I. E. (2008). Structure and Function of Claudins. Biochim. Biophys. Acta (Bba) - Biomembranes 1778 (3), 631–645. 10.1016/j.bbamem.2007.10.018 18036336

[B22] Larkin J. Chiarion-Sileni V. Gonzalez R. Grob J. J. Cowey C. L. Lao C. D. (2015). Combined Nivolumab and Ipilimumab or Monotherapy in Untreated Melanoma. N. Engl. J. Med. 373 (1), 23–34. 10.1056/NEJMoa1504030 26027431 PMC5698905

[B23] Li T. Fu J. Zeng Z. Cohen D. Li J. Chen Q. (2020a). TIMER2.0 for Analysis of Tumor-Infiltrating Immune Cells. Nucleic Acids Res. 48 (W1), W509–w514. 10.1093/nar/gkaa407 32442275 PMC7319575

[B24] Li Z. Xuan W. Huang L. Chen N. Hou Z. Lu B. (2020b). Claudin 10 Acts as a Novel Biomarker for the Prognosis of Patients with Ovarian Cancer. Oncol. Lett. 20 (1), 373–381. 10.3892/ol.2020.11557 PMC7285858 32565963

[B25] Markov A. G. (2013). Claudins as Tight junction Proteins: the Molecular Element of Paracellular Transport. Ross Fiziol Zh Im I M Sechenova 99 (2), 175–195. 23650732

[B26] Martin T. A. Jiang W. G. (2009). Loss of Tight junction Barrier Function and its Role in Cancer Metastasis. Biochim. Biophys. Acta (Bba) - Biomembranes 1788 (4), 872–891. 10.1016/j.bbamem.2008.11.005 19059202

[B27] Mereiter S. Balmaña M. Gomes J. Magalhães A. Reis C. A. (2016). Glycomic Approaches for the Discovery of Targets in Gastrointestinal Cancer. Front. Oncol. 6, 55. 10.3389/fonc.2016.00055 27014630 PMC4783390

[B28] Mostafavi S. Morris Q. (2012). Combining many Interaction Networks to Predict Gene Function and Analyze Gene Lists. Proteomics 12 (10), 1687–1696. 10.1002/pmic.201100607 22589215

[B29] Motzer R. J. Tannir N. M. McDermott D. F. Arén Frontera O. Melichar B. Choueiri T. K. (2018). Nivolumab Plus Ipilimumab versus Sunitinib in Advanced Renal-Cell Carcinoma. N. Engl. J. Med. 378 (14), 1277–1290. 10.1056/NEJMoa1712126 29562145 PMC5972549

[B30] Overman M. J. Lonardi S. Wong K. Y. M. Lenz H.-J. Gelsomino F. Aglietta M. (2018). Durable Clinical Benefit with Nivolumab Plus Ipilimumab in DNA Mismatch Repair-Deficient/Microsatellite Instability-High Metastatic Colorectal Cancer. Jco 36 (8), 773–779. 10.1200/jco.2017.76.9901 29355075

[B31] Rawla P. Barsouk A. (2019). Epidemiology of Gastric Cancer: Global Trends, Risk Factors and Prevention. pg 14 (1), 26–38. 10.5114/pg.2018.80001 PMC6444111 30944675

[B32] Rhodes D. R. Kalyana-Sundaram S. Mahavisno V. Varambally R. Yu J. Briggs B. B. (2007). Oncomine 3.0: Genes, Pathways, and Networks in a Collection of 18,000 Cancer Gene Expression Profiles. Neoplasia 9 (2), 166–180. 10.1593/neo.07112 17356713 PMC1813932

[B33] Sahin U. Manikhas G. Lordick F. Rusyn A. Vynnychenko I. Dudov A. (2021). FAST: a Randomised Phase II Study of Zolbetuximab (IMAB362) Plus EOX versus EOX Alone for First-Line Treatment of Advanced CLDN18.2-positive Gastric and Gastro-Oesophageal Adenocarcinoma. Ann. Oncol. 32 (5), 609–619. 10.1016/j.annonc.2021.02.005 33610734

[B34] Sanada Y. Oue N. Mitani Y. Yoshida K. Nakayama H. Yasui W. (2006). Down-regulation of the Claudin-18 Gene, Identified through Serial Analysis of Gene Expression Data Analysis, in Gastric Cancer with an Intestinal Phenotype. J. Pathol. 208 (5), 633–642. 10.1002/path.1922 16435283

[B35] Sawada N. Murata M. Kikuchi K. Osanai M. Tobioka H. Kojima T. (2003). Tight Junctions and Human Diseases. Med. Electron Microsc. 36 (3), 147–156. 10.1007/s00795-003-0219-y 14505058

[B36] Singh P. Toom S. Huang Y. (2017). Anti-claudin 18.2 Antibody as New Targeted Therapy for Advanced Gastric Cancer. J. Hematol. Oncol. 10 (1), 105. 10.1186/s13045-017-0473-4 28494772 PMC5427576

[B37] Stuart D. Sellers W. R. (2009). Linking Somatic Genetic Alterations in Cancer to Therapeutics. Curr. Opin. Cel Biol. 21 (2), 304–310. 10.1016/j.ceb.2009.02.001 19328671

[B38] Swisshelm K. Macek R. Kubbies M. (2005). Role of Claudins in Tumorigenesis. Adv. Drug Deliv. Rev. 57 (6), 919–928. 10.1016/j.addr.2005.01.006 15820559

[B39] Szász A. M. Lánczky A. Nagy Á. Busuttil A. S. Hark K. Green J. E. (2016). Cross-validation of Survival Associated Biomarkers in Gastric Cancer Using Transcriptomic Data of 1,065 Patients. Oncotarget 7 (31), 49322–49333. 10.18632/oncotarget.10337 27384994 PMC5226511

[B40] Tabariès S. Siegel P. M. (2017). The Role of Claudins in Cancer Metastasis. Oncogene 36 (9), 1176–1190. 10.1038/onc.2016.289 27524421

[B41] Tang Z. Kang B. Li C. Chen T. Zhang Z. (2019). GEPIA2: an Enhanced Web Server for Large-Scale Expression Profiling and Interactive Analysis. Nucleic Acids Res. 47 (W1), W556–w560. 10.1093/nar/gkz430 31114875 PMC6602440

[B42] Uhlen M. Oksvold P. Fagerberg L. Lundberg E. Jonasson K. Forsberg M. (2010). Towards a Knowledge-Based Human Protein Atlas. Nat. Biotechnol. 28 (12), 1248–1250. 10.1038/nbt1210-1248 21139605

[B43] van Dijk N. Gil-Jimenez A. Silina K. Hendricksen K. Smit L. A. de Feijter J. M. (2020). Preoperative Ipilimumab Plus Nivolumab in Locoregionally Advanced Urothelial Cancer: the NABUCCO Trial. Nat. Med. 26 (12), 1839–1844. 10.1038/s41591-020-1085-z 33046870

[B44] Vasaikar S. V. Straub P. Wang J. Zhang B. (2018). LinkedOmics: Analyzing Multi-Omics Data within and across 32 Cancer Types. Nucleic Acids Res. 46 (D1), D956–d963. 10.1093/nar/gkx1090 29136207 PMC5753188

[B45] Warde-Farley D. Donaldson S. L. Comes O. Zuberi K. Badrawi R. Chao P. (2010). The GeneMANIA Prediction Server: Biological Network Integration for Gene Prioritization and Predicting Gene Function. Nucleic Acids Res. 38, W214–W220. 10.1093/nar/gkq537 20576703 PMC2896186

[B46] Xiang Z. Zhong C. Chang A. Ling J. Zhao H. Zhou W. (2020). Immune-related Key Gene CLDN10 Correlates with Lymph Node Metastasis but Predicts Favorable Prognosis in Papillary Thyroid Carcinoma. Aging 12 (3), 2825–2839. 10.18632/aging.102780 32045884 PMC7041783

[B47] Yang W. Li L. Zhang K. Ma K. Gong Y. Zhou J. (2021). CLDN10 Associated with Immune Infiltration Is a Novel Prognostic Biomarker for clear Cell Renal Cell Carcinoma. Epigenomics 13, 31–45. 10.2217/epi-2020-0256 33203244

[B48] Zhang Z. Wang A. Sun B. Zhan Z. Chen K. Wang C. (2013). Expression of CLDN1 and CLDN10 in Lung Adenocarcinoma *In Situ* and Invasive Lepidic Predominant Adenocarcinoma. J. Cardiothorac. Surg. 8, 95. 10.1186/1749-8090-8-95 23591077 PMC3639873

