# Supplementary material for: Down-Regulated CLDN10 Predicts Favorable Prognosis and Correlates With Immune Infiltration in Gastric Cancer
Source: Front Genet. 2021 Oct 13;12:747581. doi: 10.3389/fgene.2021.747581 (PMC8548647; doi:10.3389/fgene.2021.747581)
Supplement: Supplementary file 2 [file Table1.DOCX]

|  | **OS** **PFS** | | | | | | | | | | | |
| --- | --- | --- | --- | --- | --- | --- | --- | --- | --- | --- | --- | --- |
|  | *N* | | *HR* | *p value* | |  | | *N* | | *HR* | | *p* Value |
| **Sex** |  |  | | |  | |  | |  | |  | |
| Female | 236 | 2.18(1.46-3.28) | | | **0.0001** | | 201 | | 1.98(1.31-2.99) | | **0.00095** | |
| male | 544 | 1.64(1.32-2.04) | | | **5.3544e-6** | | 437 | | 1.61(1.21-2.15) | | **0.00092** | |
| **Stage** |  |  | | |  | |  | |  | |  | |
| 1 | 67 | 3.22(1.11-9.32) | | | **0.023** | | 60 | | 2.37(0.77-7.3) | | 0.12 | |
| 2 | 140 | 2.55(1.33-4.88) | | | **0.0034** | | 131 | | 2.42(1.32-4.42) | | **0.0031** | |
| 3 | 305 | 1.7(1.27-2.27) | | | **3e-04** | | 186 | | 0.8(0.53-1.23) | | 0.31 | |
| 4 | 148 | 1.66(1.07-2.59) | | | **0.023** | | 141 | | 1.54(0.96-2.46) | | 0.073 | |
| **Stage T** |  |  | | |  | |  | |  | |  | |
| 2 | 241 | 1.56(1.02-2.39) | | | **0.037** | | 239 | | 1.59(1.05-2.41) | | **0.027** | |
| 3 | 204 | 0.83(0.58-1.19) | | | 0.31 | | 204 | | 0.77(0.55-1.1) | | 0.15 | |
| 4 | 38 | 0.58(0.23-1.5) | | | 0.26 | | 39 | | 0.61(0.24-1.51) | | 0.28 | |
| **Stage N** |  |  | | |  | |  | |  | |  | |
| 0 | 74 | 3.06(1.25-7.48) | | | **0.01** | | 72 | | 2.96(1.21-7.22) | | **0.013** | |
| 1 | 225 | 2.01(1.26-3.2) | | | **0.0028** | | 222 | | 1.84(1.18-2.88) | | **0.0063** | |
| 2 | 121 | 0.88(0.54-1.43) | | | 0.6 | | 125 | | 0.74(0.48-1.13) | | 0.16 | |
| 3 | 76 | 1.82(1.03-3.22) | | | **0.036** | | 76 | | 1.92(1.05-3.52) | | **0.032** | |
| 1+2+3 | 422 | 1.33(1.02-1.74) | | | **0.034** | | 423 | | 1.28(0.99-1.65) | | **0.059** | |
| **Lauren classification** |  |  | | |  | |  | |  | |  | |
| intestinal | 320 | 2.15(1.55-2.98) | | | **2.3e-06** | | 263 | | 1.6(1.13-2.27) | | **0.0079** | |
| diffuse | 241 | 1.28(0.89-1.83) | | | 0.1 | | 231 | | 1.18（0.82-1.69） | | 0.37 | |
| mixed | 32 | 1.71(0.54-5.38) | | | 0.36 | | 28 | | 0.43（0.15-1.21） | | **0.099** | |
| **Treatment** |  |  | | |  | |  | |  | |  | |
| surgery alone | 380 | 1.54(1.08-2.19) | | | **0.017** | | 375 | | 1.43（1.01-2.02） | | **0.044** | |
| 5 FU based adjuvant | 152 | 0.74(0.5-1.08) | | | 0.12 | | 152 | | 0.73（0.49-1.08） | | 0.12 | |
| other adjuvant | 86 | 0.37(0.15-0.89) | | | **0.021** | | 80 | | 0.42（0.19-0.93） | | **0.028** | |
| **HER2** |  |  | | |  | |  | |  | |  | |
| negative | 532 | 1.8(1.44-2.26) | | | **2.1e-07** | | 408 | | 1.62（1.24-2.12） | | **0.00033** | |
| positive | 343 | 1.25(0.95-1.64) | | | 0.11 | | 232 | | 1.31（0.9-1.92） | | 0.16 | |
| **Stage M** |  |  | | |  | |  | |  | |  | |
| 0 | 444 | 1.42（1.07-1.87） | | | **0.013** | | 443 | | 1.35（1.03-1.76） | | **0.027** | |
| 1 | 56 | 2（1.08-3.71） | | | **0.024** | | 56 | | 1.82（0.97-3.39） | | 0.058 | |
