# Supplementary material for: Down-Regulated CLDN10 Predicts Favorable Prognosis and Correlates With Immune Infiltration in Gastric Cancer
Source: Front Genet. 2021 Oct 13;12:747581. doi: 10.3389/fgene.2021.747581 (PMC8548647; doi:10.3389/fgene.2021.747581)
Supplement: Supplementary file 3 [file Table2.DOCX]

| **Description** | **Gene markers** |  |  |  | **STAD** |  |  |  |  |
| --- | --- | --- | --- | --- | --- | --- | --- | --- | --- |
|  |  |  | None |  |  |  | Purity |  |  |
|  |  | Cor |  | *P* |  | Cor |  | *P* |  |
| CD8 + T cell | CD8A | -0.063 |  | 0.198 |  | -0.077 |  | 0.133 |  |
|  | CD8B | 0.054 |  | 0.274 |  | 0.058 |  | 0.262 |  |
| T cell (general) | CD3D | -0.083 |  | 0.0899 |  | -0.116 |  | **0.0245** |  |
|  | CD3E | -0.045 |  | 0.356 |  | -0.079 |  | 0.126 |  |
|  | CD2 | -0.066 |  | 0.18 |  | -0.091 |  | 0.0781 |  |
| B cell | CD19 | 0.203 |  | **3.21E-05** |  | 0.174 |  | **6.80E-04** |  |
|  | CD79A | 0.153 |  | **1.76E-03** |  | 0.127 |  | **1.34E-02** |  |
| Monocyte | CD86 | -0.059 |  | 2.32E-01 |  | -0.081 |  | 0.115 |  |
|  | CSF1R | 0.027 |  | 0.589 |  | -0.003 |  | 0.954 |  |
| TAM | CCL2 | 0.106 |  | **0.0305** |  | 0.098 |  | 0.0569 |  |
|  | CD68 | -0.065 |  | 0.19 |  | -0.068 |  | 0.189 |  |
|  | IL10 | 0.049 |  | 0.32 |  | 0.02 |  | 0.698 |  |
| M1 Macrophage | NOS2 | -0.169 |  | **5.54E-04** |  | -0.19 |  | **2.04E-04** |  |
|  |  |  |  |  |  |  |  |  |  |
|  | IRF5 | 0.083 |  | 9.18E-02 |  | 0.072 |  | 0.162 |  |
|  | PTGS2 | 0.111 |  | **2.34E-02** |  | 0.099 |  | 5.40E-02 |  |
| M2 Macrophage | CD163 | -0.032 |  | 0.519 |  | -0.047 |  | 0.362 |  |
|  | VSIG4 | -0.01 |  | 0.843 |  | -0.015 |  | 0.768 |  |
|  | MS4A4A | 0.007 |  | 0.887 |  | -0.007 |  | 0.888 |  |
| Neutrophils | CEACAM8 | 0.08 |  | 0.106 |  | 0.078 |  | 0.127 |  |
|  | ITGAM | 0.019 |  | 0.695 |  | 0.001 |  | 0.214 |  |
|  | CCR7 | 0.146 |  | **2.93E-03** |  | 0.12 |  | **1.97E-02** |  |
| Natural killer cell | KIR2DL1 | 0.019 |  | 0.698 |  | 0.013 |  | 0.795 |  |
|  | KIR2DL3 | −0.052 |  | 0.289 |  | −0.063 |  | 0.224 |  |
|  | KIR2DL4 | −0.129 |  | **8.65E-03** |  | −0.132 |  | **1.01E-02** |  |
|  | KIR3DL1 | -0.047 |  | 0.344 |  | -0.048 |  | 0.349 |  |
|  | KIR3DL2 | -0.062 |  | 0.206 |  | -0.071 |  | 0.17 |  |
|  | KIR3DL3 | −0.053 |  | 0.278 |  | −0.056 |  | 0.274 |  |
|  | KIR2DS4 | -0.087 |  | 7.65E-02 |  | −0.094 |  | 6.71E-02 |  |
| Dendritic cell | HLA-DPB1 | -0.038 |  | 0.437 |  | -0.064 |  | 0.21 |  |
|  | HLA-DQB1 | -0.079 |  | 0.11 |  | -0.103 |  | **4.49E-02** |  |
|  | HLA-DRA | -0.08 |  | 0.104 |  | -0.105 |  | **4.05E-02** |  |
|  | HLA-DPA1 | -0.052 |  | 0.29 |  | -0.076 |  | 0.141 |  |
|  | CD1C | 0.238 |  | **9.35E-07** |  | 0.219 |  | **1.65E-05** |  |
| Th1 | NRP1 | 0.124 |  | **1.17E-02** |  | 0.102 |  | **4.66E-02** |  |
|  | ITGAX | -0.008 |  | 0.878 |  | -0.031 |  | 0.547 |  |
|  | TBX21 | -0.056 |  | 0.256 |  | -0.085 |  | 9.76E-02 |  |
|  | STAT1 | -0.13 |  | **7.81E-03** |  | -0.139 |  | **6.69E-03** |  |
|  | IFNG | -0.229 |  | **2.47E-06** |  | -0.243 |  | **1.64E-06** |  |
|  | TNF | 0.021 |  | 0.677 |  | -0.01 |  | 0.845 |  |
| Th2 | GATA3 | 0.047 |  | 0.34 |  | 0.032 |  | 0.528 |  |
|  | STAT6 | 0.023 |  | 0.633 |  | 0.036 |  | 0.479 |  |
|  | STAT5A | -0.003 |  | 0.947 |  | -0.022 |  | 0.67 |  |
|  | IL13 | -0.037 |  | 0.455 |  | -0.058 |  | 0.262 |  |
| Tfh | BCL6 | 0.299 |  | **4.83E-10** |  | 0.297 |  | **3.49E-09** |  |
|  | IL21 | -0.09 |  | 6.64E-02 |  | -0.109 |  | **3.34E-02** |  |
| Th17 | STAT3 | 0.138 |  | **5.01E-03** |  | 0.131 |  | **1.05E-02** |  |
|  | IL17A | -0.052 |  | 0.292 |  | -0.064 |  | 0.215 |  |
| Treg | FOXP3 | -0.11 |  | **2.51E-02** |  | -0.132 |  | **1.03E-02** |  |
|  | CCR8 | -0.035 |  | 0.478 |  | -0.057 |  | 0.269 |  |
|  | STAT5B | 0.178 |  | **2.73E-04** |  | 0.153 |  | **2.78E-03** |  |
|  | TGFB1 | 0.104 |  | **3.33E-02** |  | 0.08 |  | 0.121 |  |
| T  cell  exhaustion | PD1(PDCD1) | -0.088 |  | 7.17E-02 |  | -0.102 |  | **4.62E-02** |  |
|  | CTLA4 | -0.106 |  | **3.02E-02** |  | -0.122 |  | **1.74E-02** |  |
|  | LAG3 | -0.151 |  | **2.09E-03** |  | -0.161 |  | **1.69E-03** |  |
|  | TIM3(HAVCR2) | -0.093 |  | 5.84E-02 |  | -0.109 |  | **3.37E-02** |  |
|  | GZMB | -0.228 |  | **2.57E-06** |  | -0.244 |  | **1.57E-06** |  |
